# New distributional records for ants and the evaluation of ant species richness and endemism patterns in Mexico

**DOI:** 10.3897/BDJ.9.e60630

**Published:** 2021-05-10

**Authors:** Mario J. Aguilar-Méndez, Madai Rosas-Mejía, Miguel Vásquez-Bolaños, Gloria Angélica González-Hernández, Milan Janda

**Affiliations:** 1 Departamento de Biología, División de Ciencias Naturales y Exactas, Universidad de Guanajuato, Guanajuato, Guanajuato, Mexico Departamento de Biología, División de Ciencias Naturales y Exactas, Universidad de Guanajuato Guanajuato, Guanajuato Mexico; 2 Instituto Politécnico Nacional (IPN), Unidad Profesional Interdisciplinaria de Ingeniería Campus Guanajuato (UPIIG), Silao de la Victoria, Guanajuato, Mexico Instituto Politécnico Nacional (IPN), Unidad Profesional Interdisciplinaria de Ingeniería Campus Guanajuato (UPIIG) Silao de la Victoria, Guanajuato Mexico; 3 Instituto de Ecología Aplicada, Universidad Autónoma de Tamaulipas, Cd. Victoria, Tamaulipas, Mexico Instituto de Ecología Aplicada, Universidad Autónoma de Tamaulipas Cd. Victoria, Tamaulipas Mexico; 4 Entomología, Departamento de Boátanica y Zoología, Centro Universitario de Ciencias Biológicas y Agropecuarias, Universidad de Guadalajara, Zapopan, Jalisco, Mexico Entomología, Departamento de Boátanica y Zoología, Centro Universitario de Ciencias Biológicas y Agropecuarias, Universidad de Guadalajara Zapopan, Jalisco Mexico; 5 Biology Centre, Academy of Sciences of the Czech Republic, Ceske Budejovice, Czech Republic Biology Centre, Academy of Sciences of the Czech Republic Ceske Budejovice Czech Republic; 6 Cátedras CONACYT, Laboratorio Nacional de Análisis y Síntesis Ecológica, ENES, Universidad Nacional Autónoma de México, Morelia, Mexico Cátedras CONACYT, Laboratorio Nacional de Análisis y Síntesis Ecológica, ENES, Universidad Nacional Autónoma de México Morelia Mexico

**Keywords:** Nearctic, Neotropical, Formicidae, distribution, regionalisation

## Abstract

**Background:**

Ants (Formicidae) in Mexico have usually been undersampled despite their ecological significance and their utility as environmental service providers and bioindicators. This study estimates the species richness and the narrow endemic species number of ants across Mexico. It also documents the presence of one species newly recorded in Mexico and 19 new state-based records of 14 species from central and north Mexico. No surveys have been performed in most of the localities where we report those records, suggesting the need for a higher sampling effort across the country.

**New information:**

We present an ant species richness estimation and a narrow endemic ant species estimation in a grid of 0.5 degrees in Mexico. *Stenamma schmitii* is recorded for the first time from Mexico. Additionally, new state-based records of *Azteca velox*, *Dorymyrmex insanus*, *Camponotus coruscus*, *Camponotus striatus*, *Formica propatula*, *Lasius latipes*, *Neivamyrmex melanocephalus*, *Neivamyrmex rugulosus*, *Syscia augustae*, *Atta texana*, *Cephalotes scutulatus*, *Crematogaster crinosa* and *Temnothorax andrei* are recorded.

## Introduction

Ants play a remarkably diverse role in ecosystems. They participate in seed dispersal, predation, pollination, soil movement, decomposition and many other processes. The study of ant diversity allows us to analyse a wide variety of ecological traits, such as habitat preferences and trophic positions, which can be used to track biotic changes and anthropogenic impact (Landsberg et al. 1999). Many features of the ant community composition (diversity, species richness, distribution, association) or a single species detection can serve as a valuable indicator for monitoring environmental changes, for instance, in the case of invasive, endangered or keystone species (Underwood and Fisher 2006).

Distribution and diversity patterns of ants are driven by the same environmental factors as most other insects, such as surface complexity, vegetation, elevational gradients, water availability and temperature (Kaspari 1993, Kaspari and Weiser 2000, Wiescher et al. 2012, Szewczyk and McCain 2016). Registering new occurrence records of a well-known taxa, such as ants, is important to determine their distribution patterns.

In Mexico, there are 895 valid extant species, classified into 11 subfamilies and 97 genera (Bolton 2020, Dáttilo et al. 2020, Vásquez-Bolaños 2015, Vásquez-Bolaños 2011). Nevertheless, the Mexican myrmecofauna is still insufficiently known across many areas. Mexican ant fauna is potentially of high importance for testing diverse biogeographical and ecological theories, as the fauna combines elements from the Nearctic and Neotropical Regions (Vásquez-Bolaños 2011).

Despite the fact that half of the land surface of Mexico has been modified (González-Abraham et al. 2015), the country still bears many diverse and pristine ecosystems, from arid deserts to rainforests. Many of these ecosystems are undersampled and the country represents an excellent opportunity to boost the already increasing number of species records, which is imperative to perform before anthropogenic disturbance of the still-preserved landscape (García-Martínez et al. 2015). Assembling distributional records is important for developing comprehensive knowledge about the target species and facilitates studies of their genetical, ecological, morphological, physiological, ethological, biogeographical or functional traits. The aim of this study is to provide new information on the distribution of 14 species, representing new state records and one new record for the country and to estimate the hotspots of ant species richness and endemism across Mexico.

We present a prospective study that could be used as a base to find potential areas of high endemicity of ants and to highlight those areas in Mexico that require a higher sampling effort, thus serving as a stepping-stone for further studies that help to increase the connectivity of the distribution of ants in Mexico.

## Materials and methods

Ant species records were retrieved from Dáttilo et al. (2020) and combined with the newly-recorded species in this study. A geometric interpolation-based approach was used to review the species richness patterns. We used the package “sf” (Pebesma 2018) in R 4.0.3 to generate a gridded (0.5 degrees) species richness map of Mexico. To adjust the species richness weighted by distances, we use a tuning value of *p* = 0.5, which combines high weights for low distances and low weights for high distances. The weighted species richness was also adjusted by a sampling effort considering a factor based on the ratio of the number of species recorded in each quadrat and the maximum species number reported for each centre of species richness in the grid. Hence, the maximum relative sampling effort in each quadrat will be closer to 1 (Raedig et al. 2010). We consider a narrow endemic species *sensu* Gentry (1986) when the species interpolated distribution range is equal to or less than five quadrats (5000 km^2^ approximately) inside the grid. This limit of maximum distribution has been previously applied to delimit insect endemism (Torres-Cambas et al. 2016, Grill et al. 2002, Mosconi et al. 2014, Bazelet et al. 2016). To estimate the distribution of the narrow endemics, we propose a factor of 0.5 as the limit of the upper number of occurrences per grid (Morawetz and Raedig 2007). To perform these estimations and map the distribution patterns, we used the R package “sperich” (Lange et al. 2012). We associated the vegetation type and land cover type per record as in Dáttilo et al. (2020) to caclculate a correlation of state-based species richness over habitat richness in R 4.0.3. We used the habitat categories from INEGI (2007) which cover all the territory of Mexico at a scale of 1:250 000 and is the result of a standardised and field verified process of the categorisation of the vegetation and land use coverage, frequently used for ecological analyses of Mexico.

Field collections were performed from March 2016 to November 2017 in 14 States of Mexico. Collection sites (Table 1) represented ecologically diverse habitats in the Nearctic and Neotropical Regions of Mexico (Morrone 2010). We aimed to retrieve abundant and/or ecologically-dominant ant species in each habitat, trying to represent different ecological preferences, life strategies and phylogenetic lineages. All samples from this study were hand-collected. The average area of the sampled localities was 12 km^2^ ± 27 km^2^. Sampling was performed for at least five hours per locality and sites were selected as far as possible from human settlements. Whenever possible, ants were collected from the same nest or at the colony foraging pathway. Specimens were preserved in molecular grade ethanol (99%). Each colony location was geopositioned by using a Garmin GPSmap 62s or by the app GPS Essentials v. 4.4.25. Habitat type associated with each sample was recorded in the field and corroborated by the vectoral metadata of vegetation and land use series IV chart (INEGI 2007). The annual mean temperature (amt) and precipitation (amp) from the climate of each locality was retrieved by the WorldClim V.2.0 database (Gotelli et al. 2011, Fick and Hijmans 2017). All environmental variables were related to the geopositioned colonies on QGIS 3.10.1.

### Ant identification

Ethanol-preserved samples were processed at the Laboratory of Molecular Ecology and Biodiversity at ENES-UNAM, Morelia, Mexico. At least one individual of each morphospecies was dry-mounted and identified with the respective taxonomic keys (Bolton et al. 2006, MacKay and Vinson 1989) and images at AntWeb (California Academy of Science 2020) were used for morphological comparisons. Species-level identification keys were used for confirming each new record, together with the confirmation by the third author using the reference collection at the entomological collection of the Center of Studies in Zoology of the University of Guadalajara, Mexico. All taxonomic categories are according to the Bolton (2020)classification system. Previous records of each species were corroborated using the Global Ant Biodiversity Informatics (GABI) database (Guénard et al. 2017), the ant diversity of the Mesoamerican corridor (Longino et al. 2016) species list, the report for Mexican ants and the recent ant records report (Dáttilo et al. 2020). Vouchers were deposited at the entomological collection of the Center of Studies in Zoology of the University of Guadalajara, Mexico.

## Taxon treatments

### Atta texana

(Buckley, 1860)

75352B7F-C0AF-57D8-975E-E19A0A56426D

#### Materials

- **Type status:** Other material. **Occurrence:** lifeStage: adult; reproductiveCondition: non-reproductive; **Taxon:** kingdom: Animalia; phylum: Arthropoda; class: Insecta; order: Hymenoptera; family: Formicidae; genus: Atta; scientificNameAuthorship: (Buckley, 1860); **Location:** country: Mexico; stateProvince: Guanajuato; locality: Santa Rosa de Lima; verbatimElevation: 2296 m; decimalLatitude: 21.12928; decimalLongitude: -101.18494; **Identification:** identifiedBy: Aguilar-Méndez M.J.; Rosas-Mejía M.; Vásquez-Bolaños M.; **Event:** samplingProtocol: Hand collecting; year: 2017; month: 5; day: 20; habitat: annual temporal and semi-permanent agriculture

#### Distribution

*Atta texana* (Buckley, 1860) were found at Santa Rosa de Lima, Guanajuato, in an oak forest with an average mean temperature (amt) of 15.31ºC and 60.41 mm/cm^2^ of annual mean precipitation (amp). The native distribution of *A. texana* is continuous from Texas to Tabasco, in addition to records from Panama and Cuba. Dry habitats, similar to those that we found in Guanajuato, are represented in such nearby States of San Luis Potosi and Durango. Even though these ants are found in dry conditions, they are also recorded in more humid habitats (Mueller et al. 2011).

#### Biology

*Atta texana* cultivate fungi as food. To maintain the fungal colony, defoliation of the nearby vegetation is needed, because the plant biomass serves as detrital substrate (Schowalter and Ring 2017). This leaf cutting ant needs suitable sites for nesting and the distribution of host plants can regulate their populations (Waller 1982). The mutualistic *Leucoagaricus gongylophorus*, associated with *A. texana*, tolerates cold more than other fungi associated with leaf-cutting ants. This could explain the northern occurrence of this species (Mueller et al. 2011).

### Azteca velox

Forel, 1899

CDE07986-FA5E-5446-B52E-23823BF6B47F

#### Materials

- **Type status:** Other material. **Occurrence:** lifeStage: adult; reproductiveCondition: non-reproductive; **Taxon:** kingdom: Animalia; phylum: Arthropoda; class: Insecta; order: Hymenoptera; family: Formicidae; genus: Azteca; scientificNameAuthorship: Forel, 1899; **Location:** country: Mexico; stateProvince: Morelos; locality: Quilamula; verbatimElevation: 1084 m; decimalLatitude: 18.5176; decimalLongitude: -99.00599; **Identification:** identifiedBy: Aguilar-Méndez M.J.; Rosas-Mejía M.; Vásquez-Bolaños M.; **Event:** samplingProtocol: Hand collecting; year: 2017; month: 6; day: 6; habitat: annual temporal and semi-permanent agriculture

#### Distribution

*Azteca velox* Forel, 1899 were found in Quilamula, Morelos, a disturbed area of annual temporal agricultural landscape with an amt of 24.38ºC and 76.91 mm/cm^2^ of amp. *Azteca velox* have a broader distribution across South America and only three States of Mexico have previous records of this species (Veracruz de la Llave, Guerrero and Nayarit). The climatic conditions are similar amongst those States and Morelos, where we found this new state-level record.

#### Biology

*Azteca velox* are commonly foraging during diurnal hours and can visit extrafloral nectaries, but are characterised as a generalised scavenger. Their polydomous nests can be found in plant cavities in seasonally dry areas, synanthropic localities and coastal zones (Longino 2007).

### Camponotus coruscus

(Smith F., 1862)

DC025D6D-A1D4-5301-BBA2-A521CA3DDE10

#### Materials

- **Type status:** Other material. **Occurrence:** lifeStage: adult; reproductiveCondition: non-reproductive; **Taxon:** kingdom: Animalia; phylum: Arthropoda; class: Insecta; order: Hymenoptera; family: Formicidae; genus: Camponotus; scientificNameAuthorship: (Smith F., 1862); **Location:** country: Mexico; stateProvince: Nuevo León; locality: Cumbres; verbatimElevation: 422.4 m; decimalLatitude: 25.44029; decimalLongitude: -100.09637; **Identification:** identifiedBy: Aguilar-Méndez M.J.; Rosas-Mejía M.; Vásquez-Bolaños M.; **Event:** samplingProtocol: Hand collecting; year: 2016; month: 9; day: 2; habitat: human settlement

#### Distribution

*Camponotus coruscus* (Smith F., 1862) were found foraging in a human settlement at the ridge of the Cumbres mountain range near Monterrey, Nuevo León. The amt at the locality is 21.3ºC and 74.4 mm/cm^2^ of amp. *Camponotus coruscus* have been found in forests and near human settlements in Costa Rica (Longino 2002). This species has been previously recorded from south and central America, from Colombia to southern Mexico. Our findings expand their known distribution range by 139 km to the north.

### Camponotus striatus

(Smith F., 1862)

547A029B-67DF-536E-B934-BFD6E9FFF248

#### Materials

- **Type status:** Other material. **Occurrence:** lifeStage: adult; reproductiveCondition: non-reproductive; **Taxon:** kingdom: Animalia; phylum: Arthropoda; class: Insecta; order: Hymenoptera; family: Formicidae; genus: Camponotus; scientificNameAuthorship: Smith F., 1862); **Location:** country: Mexico; stateProvince: Nuevo León; locality: Cumbres (Estanzuela); verbatimElevation: 628.7 m; decimalLatitude: 25.55598; decimalLongitude: -100.26521; **Identification:** identifiedBy: Aguilar-Méndez M.J.; Rosas-Mejía M.; Vásquez-Bolaños M.; **Event:** samplingProtocol: Hand collecting; year: 2016; month: 9; day: 1; habitat: submontane shrubland

#### Distribution

*Camponotus striatus* (Smith F., 1862) were found in the Estanzuela locality of the Cumbres mountain system near Monterrey, Nuevo León (amt is 20.3ºC and 55.6 mm/cm^2^ of amp) in a submontane shrubland. The species are distributed throughout Central and South America. In Mexico, they can be found from the Yucatán Peninsula to Tamaulipas, including in the Pacific coastal States of Jalisco and Nayarit.

#### Biology

*Camponotus striatus* has been found nesting inside logs and twigs in forests and coffee plantations in Chiapas at altitudes ranging from 650-900 m a.s.l. (De la Mora et al. 2015).

### Cephalotes scutulatus

(Smith F., 1867)

018079F6-8637-53FA-A987-4F0F832AFCD1

#### Materials

- **Type status:** Other material. **Occurrence:** lifeStage: adult; reproductiveCondition: non-reproductive; **Taxon:** kingdom: Animalia; phylum: Arthropoda; class: Insecta; order: Hymenoptera; family: Formicidae; genus: Cephalotes; scientificNameAuthorship: (Smith F., 1867); **Location:** country: Mexico; stateProvince: Puebla; locality: Yohualichan; verbatimElevation: 615 m; decimalLatitude: 20.061324; decimalLongitude: -97.503212; **Identification:** identifiedBy: Aguilar-Méndez M.J.; Rosas-Mejía M.; Vásquez-Bolaños M.; **Event:** samplingProtocol: Hand collecting; year: 2016; month: 7; day: 4; habitat: induced grassland

#### Distribution

*Cephalotes scutulatus* (Smith F., 1867) have a known distribution that goes all the way from Colombia to the State of Texas in the U.S. In Mexico, they are recorded from several States, some of which border the State of Puebla. This solitary ant was found foraging on a tree in the archaeological zone of Yohualichan, Puebla, at 21.36ºC of amt and 172.25 mm/cm^2^ of amp.

### Crematogaster crinosa

Mayr, 1862

29B849A6-A25C-5A8D-8051-7B14D6169AA9

#### Materials

- **Type status:** Other material. **Occurrence:** lifeStage: adult; reproductiveCondition: non-reproductive; **Taxon:** kingdom: Animalia; phylum: Arthropoda; class: Insecta; order: Hymenoptera; family: Formicidae; genus: Crematogaster; scientificNameAuthorship: Mayr, 1862; **Location:** country: Mexico; stateProvince: Nuevo León; locality: Cumbres (Las Adjuntas); verbatimElevation: 723 m; decimalLatitude: 25.29747; decimalLongitude: -100.13781; **Identification:** identifiedBy: Aguilar-Méndez M.J.; Rosas-Mejía M.; Vásquez-Bolaños M.; **Event:** samplingProtocol: Hand collecting; year: 2016; month: 9; day: 1; habitat: pine-oak forest

#### Distribution

*Crematogaster crinosa* Mayr, 1862 is an ant species with a wide distribution in America, from Argentina to the State of Colorado in the U.S. Distribution in Mexico is recorded for more than half of the States including San Luis Potosí and Tamaulipas, both of which border the Nuevo León State, where we newly record their presence. *Crematogaster crinosa* was found in a pine-oak forest of Las Adjuntas locality at Cumbres mountain system in Monterrey, Nuevo León (amt is 19.3ºC and 61.8 mm/cm^2^ of amp).

#### Biology

*Crematogaster crinosa* can be found commonly in seasonally dry areas, but also in the high canopy or disturbed areas of wet forests, due to their preference for highly isolated areas. These ants can also dominate the ant population in mangroves. They are considered an omnivorous species. *C. crinosa* have been reported scavenging for insects, visiting extrafloral nectarines and tending scale insects (Longino 2003).

### Dorymyrmex insanus

(Buckley, 1866)

27EC4785-13C5-5A7A-8E15-08AD33CD98EE

#### Materials

- **Type status:** Other material. **Occurrence:** lifeStage: adult; reproductiveCondition: non-reproductive; **Taxon:** kingdom: Animalia; phylum: Arthropoda; class: Insecta; order: Hymenoptera; family: Formicidae; genus: Dorymyrmex; scientificNameAuthorship: (Buckley, 1866); **Location:** country: Mexico; stateProvince: Guanajuato; locality: Cerro Culiacán; verbatimElevation: 2085 m; decimalLatitude: 20.32546; decimalLongitude: -100.997002; **Identification:** identifiedBy: Aguilar-Méndez M.J.; Rosas-Mejía M.; Vásquez-Bolaños M.; **Event:** samplingProtocol: Hand collecting; year: 2017; month: 5; day: 13; habitat: secondary arboreal vegetation of a deciduous forest
- **Type status:** Other material. **Occurrence:** lifeStage: adult; reproductiveCondition: non-reproductive; **Taxon:** kingdom: Animalia; phylum: Arthropoda; class: Insecta; order: Hymenoptera; family: Formicidae; genus: Dorymyrmex; scientificNameAuthorship: Buckley (1866); **Location:** country: Mexico; stateProvince: Guanajuato; locality: Calderones; verbatimElevation: 2344 m; decimalLatitude: 20.989226; decimalLongitude: -101.23634; **Identification:** identifiedBy: Aguilar-Méndez M.J.; Rosas-Mejía M.; Vásquez-Bolaños M.; **Event:** samplingProtocol: Hand collecting; year: 2017; month: 9; day: 23; habitat: natural grassland
- **Type status:** Other material. **Occurrence:** lifeStage: adult; reproductiveCondition: non-reproductive; **Taxon:** kingdom: Animalia; phylum: Arthropoda; class: Insecta; order: Hymenoptera; family: Formicidae; genus: Dorymyrmex; scientificNameAuthorship: Buckley (1866); **Location:** country: Mexico; stateProvince: Guanajuato; locality: Chichindaro; verbatimElevation: 2313 m; decimalLatitude: 21.022; decimalLongitude: -101.224; **Identification:** identifiedBy: Aguilar-Méndez M.J.; Rosas-Mejía M.; Vásquez-Bolaños M.; **Event:** samplingProtocol: Hand collecting; year: 2017; month: 8; day: 13; habitat: induced grassland
- **Type status:** Other material. **Occurrence:** lifeStage: adult; reproductiveCondition: non-reproductive; **Taxon:** kingdom: Animalia; phylum: Arthropoda; class: Insecta; order: Hymenoptera; family: Formicidae; genus: Dorymyrmex; scientificNameAuthorship: Buckley (1866); **Location:** country: Mexico; stateProvince: Guanajuato; locality: Cerro de la Bufa; verbatimElevation: 2160 m; decimalLatitude: 20.999888; decimalLongitude: -101.249285; **Identification:** identifiedBy: Aguilar-Méndez M.J.; Rosas-Mejía M.; Vásquez-Bolaños M.; **Event:** samplingProtocol: Hand collecting; year: 2017; month: 9; day: 22; habitat: natural grassland

#### Distribution

*Dorymyrmex insanus* (Buckley, 1866) were found in four different localities in the Guanajuato State: Cerro Culiacan (secondary arboreal vegetation of a deciduous forest, 18.3ºC of amt and 56.25 mm/cm^2^ of amp), Calderones (natural grassland, 18.4ºC of amt and 54.75 mm/cm^2^ of amp), Chichindaro (induced grassland, 15.3ºC of amt and 60.4 mm/cm^2^ of amp) and Cerro la Bufa (natural grassland, 18.4ºC of amt and 54.75 mm/cm^2^ of amp). *Dorymyrmex insanus* are previously recorded from Colombia to Wyoming, U.S. (Guénard et al. 2017) and, in Mexico, are recorded in almost three quarters of the States.

#### Biology

Nests can be found in open areas, mounds and in temporal cultivated areas. Altitude range is from 75 to 2590 m a.s.l. (Allred 1982, Cuezzo and Guerrero 2012).

### Formica propatula

Francoeur, 1973

B5196199-599C-58C4-AEF5-9AD4C767FE51

#### Materials

- **Type status:** Other material. **Occurrence:** lifeStage: adult; reproductiveCondition: non-reproductive; **Taxon:** kingdom: Animalia; phylum: Arthropoda; class: Insecta; order: Hymenoptera; family: Formicidae; genus: Formica; scientificNameAuthorship: Francoeur 1973; **Location:** country: Mexico; stateProvince: Michoacán; locality: Encinar Quiroga; verbatimElevation: 2431 m; decimalLatitude: 19.701824; decimalLongitude: -101.468782; **Identification:** identifiedBy: Aguilar-Méndez M.J.; Rosas-Mejía M.; Vásquez-Bolaños M.; **Event:** samplingProtocol: Hand collecting; year: 2017; month: 8; day: 26; habitat: oak forest

#### Distribution

*Formica propatula* Francoeur, 1973 were found in an oak forest near Quiroga, Michoacán de Ocampo with 16ºC of amt and 67.4 mm/cm^2^ of amp. *Formica propulata* have been exclusively recorded in Mexico in Oaxaca, Guanajuato, Guerrero, Puebla, Tlaxcala, Hidalgo, Mexico State, Mexico City and Coahuila. Recently, *F. propatula* has been found associated with a template oak forest and agricultural land in Tlaxcala (Cuautle et al. 2020).

### Lasius latipes

(Walsh, 1863)

20088AD6-B74F-5752-85BF-510F06DA06F2

#### Materials

- **Type status:** Other material. **Occurrence:** lifeStage: adult; reproductiveCondition: non-reproductive; **Taxon:** kingdom: Animalia; phylum: Arthropoda; class: Insecta; order: Hymenoptera; family: Formicidae; genus: Lasius; scientificNameAuthorship: (Walsh, 1863); **Location:** country: Mexico; stateProvince: Guanajuato; locality: Rancho del Coporo; verbatimElevation: 2462; decimalLatitude: 21.438212; decimalLongitude: -101.406007; **Identification:** identifiedBy: Aguilar-Méndez M.J.; Rosas-Mejía M.; Vásquez-Bolaños M.; **Event:** samplingProtocol: Hand collecting; year: 2017; month: 10; day: 12; habitat: oak forest
- **Type status:** Other material. **Occurrence:** lifeStage: adult; reproductiveCondition: non-reproductive; **Taxon:** kingdom: Animalia; phylum: Arthropoda; class: Insecta; order: Hymenoptera; family: Formicidae; genus: Lasius; scientificNameAuthorship: Walsh (1863); **Location:** country: Mexico; stateProvince: Queretaro; locality: Jalpan de la Sierra; verbatimElevation: 2575; decimalLatitude: 21.119768; decimalLongitude: -99.659891; **Identification:** identifiedBy: Aguilar-Méndez M.J.; Rosas-Mejía M.; Vásquez-Bolaños M.; **Event:** samplingProtocol: Hand collecting; year: 2017; month: 10; day: 23; habitat: induced grassland

#### Distribution

*Lasius latipes* (Walsh, 1863) were found in an oak forest near El Coporo, Guanajuato at 15.3^0^C of amt and 46.2 mm/cm^2^ of amp and in an induced grassland in Jalpan de la Sierra, Queretaro at 18.3ºC of amt and 45.5 mm/cm^2^ of amp. *Lasius latipes* has been recorded in several States of Canada and U.S. (Guénard et al. 2017). In Mexico, this species was previously recorded only for Tamaulipas, Sonora and Tlaxcala States.

#### Biology

Colonies of *L. latipes* are strictly underground most of their lifetime. Nests are often found in sandy areas, open grassy areas, in the borders or clearings of woods of scrub oak, pine and cedar at 2200 m a.s.l. (Wing 1968)

### Neivamyrmex melanocephalus

(Emery, 1895)

A0294EAC-8508-5C5D-AF36-BB9D545ACF77

#### Materials

- **Type status:** Other material. **Occurrence:** lifeStage: adult; reproductiveCondition: non-reproductive; **Taxon:** kingdom: Animalia; phylum: Arthropoda; class: Insecta; order: Hymenoptera; family: Formicidae; genus: Neivamyrmex; scientificNameAuthorship: (Emery, 1895); **Location:** country: Mexico; stateProvince: Guanajuato; locality: Las Palomas; verbatimElevation: 2405 m; decimalLatitude: 21.0684; decimalLongitude: -101.22427; **Identification:** identifiedBy: Aguilar-Méndez M.J.; Rosas-Mejía M.; Vásquez-Bolaños M.; **Event:** samplingProtocol: Hand collecting; year: 2016; month: 10; day: 1; habitat: oak Forest

#### Distribution

*Neivamyrmex melanocephalus* (Emery, 1895) were found in an oak forest in Las Palomas, Guanajuato at 15.3ºC of amt and 60.4 mm/cm^2^ of amp. *Neivamyrmex melanocephalus* has been previously recorded in Costa Rica, Honduras, Guatemala Mexico and U.S. (Guénard et al. 2017). This species has been recorded in Michoacán de Ocampo, Jalisco and Queretaro, States that border Guanajuato, where we are reporting for the first time their presence.

#### Biology

As many members from the subfamily Dorylinae, *N. melanocephalus* forms foraging raids attacking a variety of small arthropods (Snelling and Snelling 2007).

### Neivamyrmex rugulosus

Borgmeier, 1953

BE567084-19F6-57F0-974B-207998DF73A8

#### Materials

- **Type status:** Other material. **Occurrence:** lifeStage: adult; reproductiveCondition: non-reproductive; **Taxon:** kingdom: Animalia; phylum: Arthropoda; class: Insecta; order: Hymenoptera; family: Formicidae; genus: Neivamyrmex; scientificNameAuthorship: Borgmeier, 1953; **Location:** country: Mexico; stateProvince: Guanajuato; locality: Las Palomas; verbatimElevation: 2387 m; decimalLatitude: 21.06221; decimalLongitude: -101.22733; **Identification:** identifiedBy: Aguilar-Méndez M.J.; Rosas-Mejía M.; Vásquez-Bolaños M.; **Event:** samplingProtocol: Hand collecting; year: 2016; month: 10; day: 1; habitat: oak Forest

#### Distribution

*Neivamyrmex rugulosus* Borgmeier, 1953 were found foraging in the same locality as *N. melanocephalus* (Las Palomas, Guanajuato). This species has only been recorded for Mexico and U.S. In Mexico, records come from for the same States as *N. melanocephalus*. *Neivamyrmex rugulosus* has been reported to predate other ants, such as *Trachymyrmex arizonensis* and *Pheidole desertorum*. *N. rugulosus* is reported at 1500 m a.s.l. (Snelling and Snelling 2007).

### Syscia augustae

(Wheeler, W.M., 1902)

180CF0DA-DAD9-546A-B606-0CC69B5B8A3E

#### Materials

- **Type status:** Other material. **Occurrence:** lifeStage: adult; reproductiveCondition: non-reproductive; **Taxon:** kingdom: Animalia; phylum: Arthropoda; class: Insecta; order: Hymenoptera; family: Formicidae; genus: Syscia; scientificNameAuthorship: (Wheeler, W.M., 1902); **Location:** country: Mexico; stateProvince: Queretaro; locality: Sierra Gorda; verbatimElevation: 2575 m; decimalLatitude: 21.11977; decimalLongitude: -99.6599; **Identification:** identifiedBy: Aguilar-Méndez M.J.; Rosas-Mejía M.; Vásquez-Bolaños M.; **Event:** samplingProtocol: Hand collecting; year: 2017; month: 11; day: 21; habitat: induced grassland

#### Distribution

*Syscia augustae* (Wheeler W.M., 1902) were found on an induced grassland in Jalpan De La Sierra, Queretaro, at 2575 m a.s.l., 18.3ºC of amt and 45.5 mm/cm^2^ of amp. This species has been recorded in all the U.S. southern border States. In Mexico, they are recorded in Baja California Peninsula, Sonora, Sinaloa, Nuevo León, Tamaulipas, Veracruz de Ignacio de la Llave and Oaxaca. Recently, Borowiec (2016) recorded this species in southeast Asia, in Borneo, Japan and India.

#### Biology

*Syscia augustae* is a subterranean, blind ant with predatory behaviour (Wheeler 1902). These ants can be found in the leaf litter, under stones and in branches on the soil, usually in moist habitats (MacKay and Mackay 2002).

### Temnothorax andrei

(Emery, 1895)

3EAFEDCE-A8FF-5B36-8B4B-CD5CB7FF2994

#### Materials

- **Type status:** Other material. **Occurrence:** lifeStage: adult; reproductiveCondition: non-reproductive; **Taxon:** kingdom: Animalia; phylum: Arthropoda; class: Insecta; order: Hymenoptera; family: Formicidae; genus: Temnothorax; scientificNameAuthorship: (Emery, 1895); **Location:** country: Mexico; stateProvince: Guanajuato; locality: Rancho Coporo; verbatimElevation: 2292 m; decimalLatitude: 21.34138; decimalLongitude: -101.372; **Identification:** identifiedBy: Aguilar-Méndez M.J.; Rosas-Mejía M.; Vásquez-Bolaños M.; **Event:** samplingProtocol: Hand collecting; year: 2017; month: 8; day: 13; habitat: secondary shrubland associated to an oak forest
- **Type status:** Other material. **Occurrence:** lifeStage: adult; reproductiveCondition: non-reproductive; **Taxon:** kingdom: Animalia; phylum: Arthropoda; class: Insecta; order: Hymenoptera; family: Formicidae; genus: Temnothorax; scientificNameAuthorship: Emery (1895); **Location:** country: Mexico; stateProvince: Jalisco; locality: Cerro de la Mesa; verbatimElevation: 2054 m; decimalLatitude: 21.28364; decimalLongitude: -102.011; **Identification:** identifiedBy: Aguilar-Méndez M.J.; Rosas-Mejía M.; Vásquez-Bolaños M.; **Event:** samplingProtocol: Hand collecting; year: 2017; month: 8; day: 5; habitat: secondary shrubland associated to a deciduous forest

#### Distribution

*Temnothorax andrei* (Emery, 1895) were found in two localities of Mexico: in a secondary shrubland associated with an oak forest near Coporo, Guanajuato at 15.3^0^C of amt and 46.25 mm/cm^2^ of amp and in a secondary shrubland associated with a deciduous forest on the top of a table-top mountain in Lagos de Moreno, Jalisco at 17.96ºC of amt and 53.4 mm/cm^2^ of amp. This species has only been previously recorded in Baja California Peninsula in Mexico and in the western U.S., including all border States with Mexico.

#### Biology

*Temnothorax andrei* occupy dry habitats, such as oak woodlands, coniferous forests, laurel forests, pinyon-juniper and cool deserts (Mackay 2000). Nests can be found under stones or in the spaces between rocks (Cole 1958).

### Stenamma schmittii

Wheeler, 1903

7229CFAD-545D-540E-9A3B-2FA3913DC616

#### Materials

- **Type status:** Other material. **Occurrence:** lifeStage: adult; reproductiveCondition: non-reproductive; **Taxon:** kingdom: Animalia; phylum: Arthropoda; class: Insecta; order: Hymenoptera; family: Formicidae; genus: Stenamma; scientificNameAuthorship: Wheeler, 1903; **Location:** country: Mexico; stateProvince: Guanajuato; locality: Rancho Coporo; verbatimElevation: 2292 m; decimalLatitude: 21.34138; decimalLongitude: -101.372; **Identification:** identifiedBy: Aguilar-Méndez M.J.; Rosas-Mejía M.; Vásquez-Bolaños M.; **Event:** samplingProtocol: Hand collecting; year: 2017; month: 8; day: 13; habitat: secondary shrubland vegetation associated with an oak forest

#### Distribution

*Stenamma schmittii* Wheeler, 1903 were found in a secondary shrubland vegetation associated with an oak forest near El Coporo, Guanajuato. The locality is at 15.3ºC of amt and 46.25 mm/cm^2^ of amp. This species has been previously recorded only for U.S. and Canada. Previous records showed a distribution on the north-eastern part of U.S. Our record expands their known distribution by 2,200 km.

#### Biology

*Stenamma schmittii* are predatory ants that can be found primarily in woodlands and live in dry to moist habitats. Nests are commonly found in the soil under stones, logs, rotten wood, leaf litter and other debris. Colonies have been found in altitudes from hundreds of metres to 1520 m a.s.l. (Smith 1957).

## Analysis

We report 19 new distributional records for 14 species from central and north Mexico. The record for *Stenamma schmitii* Wheeler, 1903 is the first for Mexico, while 13 of them are new state-level records. The species belong to 11 genera and four subfamilies (Dolichoderinae, Dorylinae, Formicinae and Myrmicinae).

New records were found in seven States in central Mexico and one in Nuevo Leon in the north part of the country. Most of these records (68%) were found in undisturbed habitats, such as natural grasslands, oak forests and shrublands associated with the oak forests. We also found four records in induced grasslands and one in a human settlement, A Quinta, at the ridge of the Cumbres mountain range. The localities where we found these ants were of high elevation (14 were at 2050 m a.s.l. or more) and low annual mean temperature (17.86 ±2.63^0^C) with precipitation that ranged from 172.25 to 45.5 mm/cm^2^.

**Figure 1.**
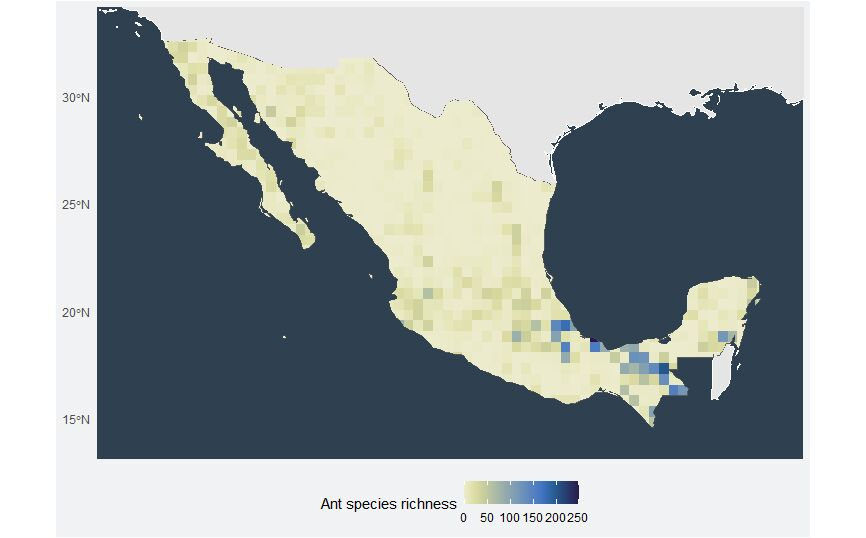
Species richness map of ants (Formicidae) in Mexico at 0.5° grids. The data are based on Dáttilo et al. (2020) records and combined with the newly-recorded species in this study. Each quadrat corresponds approximately to a size of 50 000 km^2^. Ant species richness is indicated from the minimum (yellow) to maximum (blue) of species per quadrat.

The analysis of 21,741 records of 888 species distributed in 856 quadrats of 0.5° revealed a maximum species richness of 251 with an average of 14.82 ± 27.8 species per quadrat. One third of the quadrats had no species recorded (*Fig. 1*).

**Figure 2.**
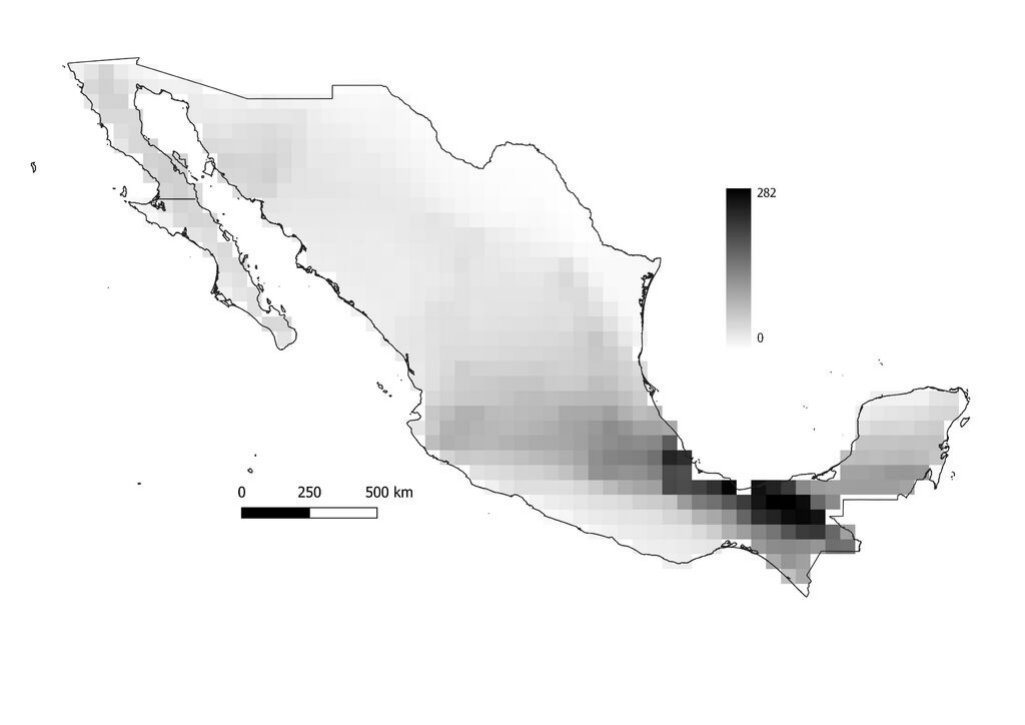
Weighted Species Richness estimation of ants in Mexico at 0.5° resolution. The maximum species number per quadrat is adjusted by sampling effort. Quadrats correspond approximately to a size of 50 000 km^2^ each. Ant species richness is classified by the quantile indicating the maximum (dark) to minimum (white) of species.

The highest ant species richness was found in the southeast region of Mexico, stretching along the southeast part of Sierra Madre Oriental and Sierra de Chiapas mountain systems. There are also two separated quadrats with high richness in central Jalisco and eastern Quintana-Roo (75 and 102 species per quadrat, respectively, Fig. 1). The quadrat with the highest species richness (251 species) is located at the southern border of the country between Tabasco and Chiapas States. However, the weighted species richness adjusted by sampling effort shows only one large area (1.14 x 105 km^2^) of high diversity with a centroid in Tabasco, Veracruz de Ignacio de la Llave and Chiapas States and a single quadrat in Quintana-Roo (Fig. 2).

Although one third of the quadrats have zero species recorded, after adjusting the species richness by sampling effort, the north-eastern border of Mexico is the only zone that lacks any recorded species (Fig. 2). Both species richness and adjusted species richness suggest the same hotspot in south-eastern Mexico. Considering the proportion of quadrats of zero species (Fig. 1), the most undersampled zones are represented by the north region of Mexico, specifically the States of Sinaloa, Coahuila, Nuevo León, Tamaulipas, Zacatecas and San Luis Potosi. There is also an undersampled region on the southern coast of Michoacán de Ocampo, Guerrero and Oaxaca.

The highest number of endemics (34) was found in the same centroid as the highest species richness, which corresponds to the Neotropical zone of Mexico. Other areas with high endemism are located the northern part of the States of Sinaloa and Baja California.

**Figure 3.**
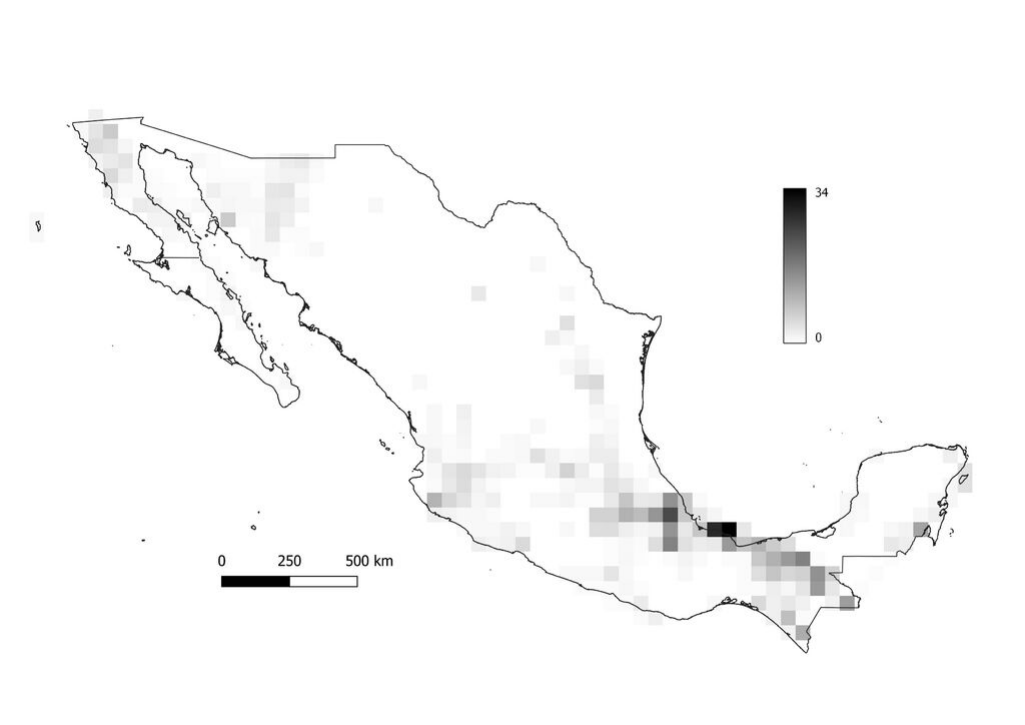
Narrow endemics of ant species in Mexico at 0.5° resolution. Endemic species richness is classified by the quantile indicating the minimum (0, white) to maximum (34, dark) narrow endemic species; white quadrats represent zero narrow endemic species.

Even though Mexico has more than 3000 islands (INEGI 2015), only 14 of them have been reported to have ant species (Dáttilo et al. 2020) and, in our analysis, three of them appeared to have a positive narrow endemic index (Guadalupe in the Pacific, Angel de la Guarda in the Sea of Cortes and Cozumel in the Caribbean, Fig. 3).

## Discussion

Six of the species recorded here (*N. melanocephalus*, *N. rugulosus*, *C. coruscus*, *C. striatus*, *C. trepidulus* and *T. andrei*) belong to the genera with the most species recorded in the country, such as *Camponotus*, which represents almost 10% of the species in Mexico (Dáttilo et al. 2020).

Half of the new reported records were collected in Guanajuato, a State which has a rather low habitat richness (number of different habitat types) and which has been a poorly-sampled region of Mexico (Table 2), with one of the lowest number of native species recorded (37). In contrast, the State of Tabasco has comparable habitat richness, but a dramatically higher number of records than Guanajuato (1251 and 176, respectively).

Alongside Guanajuato, States Michoacán de Ocampo, Aguascalientes, Zacatecas and San Luis Potosí have low numbers of ant records (Table 2). However, these States converge in a region where narrow endemic species are distributed and appear to be in an ant endemism hotspot (Fig. 3) that corresponds to the transition zone between the Nearctic and Neotropical areas of Mexico. The Mexican transition zone has been reported as a centre of endemism of other taxa (plants, mammals) and the Trans-Mexican volcanic belt could have a specific taxa with a primary biogeographic homology (cenocron) where vicariance events were initiated by the volcanic events (Halffter and Morrone 2017, Morrone 2010).

Dáttilo et al. (2020) report only 33 species in 166 records in Guanajuato within 11 different habitats (only 56% of the records have the habitat reported). In comparision, Veracruz de Ignacio de la Llave has 36 different habitat types, 4329 records and 454 species documented. Most of the narrow endemics are also found in Veracruz de Ignacio de la Llave (Fig. 3), where habitats seem to be more diverse and species richness is higher, even after adjusting the values by sampling effort (Fig. 2). This State has a unique geographical position and its species richness and diverse landscape types could be a result of the of the altitudinal gradients ranging from sea level to 5700 m a.s.l. at the Pico de Orizaba.

We have to also consider two field biological stations that have been centres of ant research in Mexico, one from the National Autonomous University of Mexico (UNAM) and the other from the Mexican Institute of Ecology (INECOL). They are located in Veracruz de la Llave and are influencing the number of records and, thereby, the endemicity indexes in their area. This can be also a factor that leads to the two separate quadrats of high richness found in Quintana-Roo and Jalisco, where entomologists from the University of Guadalajara and from the South Border College (Ecosur) conducted numerous field expeditions.

Habitat richness plays an important role in ant species distribution and should be considered when proposing new surveys. We found a significant correlation (R^2^_(30)_ = 0.55 p < 0.0001) for ant species richness and habitat richness in the state-based records of Mexico (Dáttilo et al. 2020). A similar relation (R^2^ = 0.40) was found by Ryder Wilkie et al. (2010) in Amazonian Ecuador using a multiple regression model with environmental variables and species richness.

The species richness, adjusted by sampling effort, reveals a zone of high species richness forming a corridor from central Jalisco across the border between Michoacán de Ocampo and Guanajuato and connecting to the main hotspot in Veracruz de Ignacio de la Llave, passing through the Trans-Mexican Volcanic Belt. (Fig. 2).

Ríos-Casanova (2014) indicated a direct relationship between the species richness and the area of each State, but also showed that the collection effort was higher in those States with more species recorded (Table 2Dáttilo et al. 2020). Even though our sampling was not aimed to exhaustively collect at every locality, we still found new records for the sampled States and one new record for Mexico. This suggests that there is still a great possibility to collect ants that will be new to Mexican States and a considerable number of new species may result from further surveys of the country. A survey performed in southern Mexico and Costa Rica demonstrated that the new species proportion can rise up to almost 80% of the records (Longino 2019).

One third of the quadrats of the country have no species records, and the overall data are highly scattered. The representative character of such a database is far from ideal for the size and the geographic diversity of the country. Therefore, we encourage the use of this information as a guidance for new surveys of ant diversity and focus on those areas with no or little records to improve the coverage of ant species distribution data. A particular effort could be taken to sample the northern Pacific coast of Mexico and the Sierra Madre Occidental (mainly all Sinaloa, southwest Chihuahua and northwest Durango States), where elements of the Nearctic, Neotropical and transition regions converge. This undersampled zone has an interesting west to east habitat gradient where agricultural landscapes, deciduous forests, pine and oak forests can be found (INEGI 2007). Another largely undersampled zone occurs at the east region of the northern border of Mexico, from Tamaulipas to Chihuahua and corresponds to the eastern zone of the Mexican Nearctic. This is a large area composed mainly of a large desert plateau with scattered patches of agricultural zones (INEGI 2007).

Despite the relevant ant distribution information that can be retrieved from surveys of the northern border of Mexico, Sinaloa and even the south regions of Guerrero and Oaxaca, entomologists might have avoided those areas because the lack of security for fieldwork. Surveys on those areas should be undertaken with extreme precautions due to continuous dangers for field biologists.

Hand collecting was the only method used to retrieve the specimens in our study and it is also the most frequent method amongst all the ant records for Mexico. The second most frequent methods are pitfall traps and leaf litter sampling (Winkler extraction) (Dáttilo et al. 2020). Hand collecting has to be standardised for quantitative surveys and is biased by the expertise of the field collector (Gotelli et al. 2011). However, records retrieved by this method have been useful to detect many (95%) of the endemic ant species (Salata et al. 2020). We encourage the use of a mixture of collecting methods, such as pitfall traps, Winkler extractors and baits for new surveys on the undersampled areas, but always using hand collecting as a method to improve the species records.

The distribution of cosmopolitan *Dorymyrmex insanus* could be used as an indicator for undersampled regions. According to Guénard et al. (2017), this species is present in all Mexican States, but Chiapas, Tabasco, Morelos, Colima, Guanajuato and Zacatecas. Here, we report its presence in Guanajuato, supporting the ubiquitous distribution of this ant across Mexico. Sampling efforts could be directed to those areas where this ant is not yet recorded. These ant species, considered as bioindicators, are often widely distributed and may be ecologically dominant in their respective biomes (Del Toro et al. 2012).

One of the most interesting biological phenomena of Mexico is the transition between the Nearctic and Neotropical biogeographical regions. To better understand the history of the biotic patterns this gradient has been generating, it will be interesting to focus on the comparative evolutionary history of species endemic from both areas, as well species distributed across this transition. Here, we documented that *A. texana*, *C. scutulatus*, *C. crinosa*, *D. insanus*, *N. melanocephalus* and *S. augustae* have distributions combining both biogeographic elements and could serve as useful models to study this phenomenon.

The results of the analysis of the distribution of species richness and endemicity of ants in Mexico were made using the more complete and up-to-date database of ant records in Mexico (Dáttilo et al. 2020). However, those records do not come from a standardised sampling system and the information is highly fragmented; therefore, the distribution patterns presented here can be improved. The necessary data to extend these patterns cannot be generated by a single systematic survey on ant biodiversity in the country. The integration of the generated data by surveys of different studies will be more valuable if the sampling is standardised and repeatable.

The general patterns of the preliminary estimation of endemism must be taken with caution, as the lack of information from some areas of the country might cause an overestimation of the levels of endemism in some ant species. Mexico is still a largely undersampled country for ants compared with regions, such as Florida (Ohyama et al. 2020) or Madagascar (Guénard et al. 2017), where the sampling has been more intense and systematic.

This is the first effort to describe the patterns of ants`species richness hotspots in Mexico and the endemicity patterns for a grid of 0.5° map of the country. To describe the processes that drove the distribution of the narrow endemic species of ants in the transition zone between the Nearctic and Neotropical zones in Mexico, more biogeographic studies are needed (Fisher 2009). It is necessary to generate a standardised sampling system to achieve the necessary representation of each quadrat and include sufficent ant species distribution data to explore the impact of Formicidae in the geobiotic scenarios in Mexico (Halffter and Morrone 2017).

## Supplementary Material

XML Treatment for Atta texana

XML Treatment for Azteca velox

XML Treatment for Camponotus coruscus

XML Treatment for Camponotus striatus

XML Treatment for Cephalotes scutulatus

XML Treatment for Crematogaster crinosa

XML Treatment for Dorymyrmex insanus

XML Treatment for Formica propatula

XML Treatment for Lasius latipes

XML Treatment for Neivamyrmex melanocephalus

XML Treatment for Neivamyrmex rugulosus

XML Treatment for Syscia augustae

XML Treatment for Temnothorax andrei

XML Treatment for Stenamma schmittii

## Figures and Tables

**Table 1.** Sampling distribution from the survey of this study. All records, but the reported ones are registered in Dáttilo et al. (2020). This survey was performed across the fourteen States in México from 2016 to 2019.

State	Collected colonies	Localities	Habitats
Coahuila	27	1	2
Colima	4	1	1
Guanajuato	68	8	8
Jalisco	21	3	5
Michoacán	23	5	5
Morelos	30	1	2
Nayarit	1	1	1
Nuevo León	21	5	6
Oaxaca	17	2	3
Puebla	9	5	5
Queretaro	16	2	6
Quintana Roo	22	5	5
Tamaulipas	39	2	5
Veracruz	32	3	5

**Table 2.** Habitat and species richness in the 32 States from Mexico. Habitat classification was retrieved from INEGI (2007), species richness and records were retrieved from Dáttilo et al. (2020) plus the newly-reported records on this study.

State	Records	Species Richness	Habitat Richness
Aguascalientes	39	17	4
Baja California	925	111	13
Baja California Sur	782	64	6
Campeche	227	104	6
Chiapas	6902	359	17
Chihuahua	265	48	7
Ciudad de México	40	26	5
Coahuila	77	31	13
Colima	50	24	5
Durango	287	59	15
Guanajuato	176	40	11
Guerrero	122	59	6
Hidalgo	525	80	12
Jalisco	1131	191	22
México	138	55	15
Michoacán	190	72	12
Morelos	562	88	8
Nayarit	65	28	8
Nuevo León	232	61	14
Oaxaca	504	215	17
Puebla	326	104	17
Querétaro	239	64	13
Quintana Roo	757	105	7
San Luis Potosí	217	51	18
Sinaloa	101	24	6
Sonora	621	102	16
Tabasco	1251	162	11
Tamaulipas	222	88	18
Tlaxcala	99	60	9
Veracruz	4329	454	36
Yucatán	137	40	6
Zacatecas	210	15	10
